# Metabolomics in High Grade Gliomas

**DOI:** 10.51520/2766-2586-17

**Published:** 2022-09-30

**Authors:** Nina Yu, Orwa Aboud

**Affiliations:** 1 University of California, Davis School of Medicine, Sacramento, CA, United States; 2 Department of Neurology and Neurological Surgery, University of California, Davis, Sacramento, CA, United States

**Keywords:** metabolomics, glioma, tumor, brain, cancer, CNS

## Abstract

Gliomas are central nervous system (CNS) cancers that are challenging to treat due to their high proliferation and mutation rates. High grade gliomas include grade 3 and grade 4 tumors, which characteristically have a poor prognosis despite advancements in diagnostic methods and therapeutic options. Advances in metabolomics are resulting in more insight as to how cancer modifies the metabolism of the cell and surrounding tissue. Hence, this avenue of research may also emerge as a way to precisely target metabolites unique to gliomas. These biomarkers may provide opportunities for glioma diagnosis, prognosis and future therapeutic intervention.

In this review, we harvest the literature that highlights notable biomolecules in high grade gliomas and promising therapeutic targets and interventions.

## Introduction

High-grade gliomas (HGGs) include the World Health Organization (WHO) grade 3 and 4 tumors and consist of astrocytoma (IDH-mutant), oligodendroglioma (IDH-mutant and 1p/19q-codeleted), glioblastoma (IDH-wildtype), diffuse hemispheric glioma (H3 G34-mutant and H3 K27-mutant), among other gliomas (Louis *et al*., 2021). The typical treatment plan includes surgical resection and a combination of chemotherapy and radiotherapy. Despite that, these tumors tend to recur with poor prognosis and limited treatment options.

Metabolomics is the study of endogenous metabolites and low molecular weight molecules (LMWM) and their interactions within a biological sample (Nicholson and Lindon, 2008; Patti *et al*., 2012). Metabolomics is a discipline integral to advancing scientific understanding and discovery of new, effective clinical diagnoses and interventions. The significance of this discipline has appeared in how we frame and diagnose cancers. Historically, the WHO has classified and diagnosed cancers histogenetically. More recently, in the fifth edition of the WHO classification of tumors of the Central Nervous System (WHO CNS5), cancer taxonomy and diagnosis are layered and involves an integration of molecular features and histology (Louis *et al*., 2021).

This review describes recent metabolomic studies that pertain to high grade gliomas, spanning metabolic pathways, metabolites, therapeutic interventions, and testing methods.

## Critical metabolic pathways

In normal mammalian cells, metabolic pathways maintain homeostasis and sustain life. However, cancer cells disrupt and exploit metabolism to promote abnormal growth, survival, and proliferation. The Warburg Effect is a well-documented phenomenon that illustrates metabolic dysregulation (Warburg, 1925; Warburg, 1956). Under typical conditions, glucose is oxidized to pyruvate through glycolysis and mitochondrial oxidation of pyruvate to acetyl-CoA continues to support energy production via the tricarboxylic acid cycle (TCA) and oxidative phosphorylation. In the 1920s, Otto Warburg observed that cancer cells have increased glucose uptake and produce lactate from glucose despite the presence of oxygen and completely functioning mitochondria (Warburg, 1925; Warburg, 1956).

Increased lactate generation noted in the Warburg effect can be explained, in part, by the activation of the PI3K/AKT/mTOR pathway by mutation in the tumor suppressor, PTEN, and/or epidermal growth factor (Chakravarti *et al*., 2004; Choe *et al*., 2003). In GBM, this pathway’s downstream effects include increased expression of vascular endothelial growth factor (VEGF) and the transcription factor, hypoxia-inducible factor-1a (HIF-1a) (Fischer et al., 2005). VEGF is a key mediator in GBM angiogenesis and is stimulated by hypoxia, which is prevalent in malignant neoplasms such as GBM. Hypoxia stabilizes HIF-1a which in turn increases expression of hexokinase-2 (HK2) in GBM cells that then promotes glycolysis and activation of factors that stimulate the transport of lactate to the extracellular space, and tumor growth and invasion (Wolf *et al*., 2011).

Aberrant metabolic behavior in cancer cells is also evident in glutaminolysis, the pentose phosphate pathway, and fatty oxidation. The TCA cycle occurs in the mitochondrial matrix and yields coenzymes NADH, GTP and FADH2, and intermediates including citrate, isocitrate, α-ketoglutarate (α-KG), succinyl-CoA, succinate, fumarate, malate and oxaloacetate. Cancer cells exploite enzymes and intermediates of the TCA cycle to promote tumorigenesis (Chen and Russo, 2012; Eng *et al*., 2003; Pavlova and Thompson, 2016). A well-established example in gliomas is isocitrate dehydrogenase (IDH) (Yan et al., 2009), which converts isocitrate to α-KG, mutations have also been found in succinate dehydrogenase (SDH) and fumarate hydratase (FH) in other conditions including phaeochromocytoma, paraganglioma, and renal cell carcinoma (Eng *et al*., 2003).

The TCA cycle can also operate if pertinent amino acids are available for metabolism. Through a series of steps, glutaminolysis converts glutamine to α-KG and thereby replenishes the TCA cycle. Cancer cells have been found to leverage glutaminolys is to increase production of metabolites such as α-KG and aspartate to support cell growth and division (Chen and Russo, 2012; Eagle, 1955; Wang *et al*., 2020).

The pentose phosphate pathway normally functions to generate NADPH and pentose sugars from glucose-6-phosphate (G6P). Not only is this pathway important for normal cells, but it is also pivotal in cancer cells since it provides building blocks for nucleic acid synthesis and protection from oxidative damage (Jin and Zhou, 2019; Patra and Hay, 2014).

Fatty acid oxidation occurs in multiple organelle types including the mitochondria, peroxisome, and endoplasmic reticulum (Talley and Mohiuddin, 2022). This process generates energy under stress and is linked to cellular respiration and proliferation in malignant gliomas (Lin *et al*., 2017).

Evidence of cancer’s unique energy metabolism and the role of the mitochondria have led some in the scientific community to consider cancer a metabolic disease (Erb *et al*., 2008; Khan *et al*., 2021; Kwon *et al*., 2015; Seyfried and Shelton, 2010; Spratlin *et al*., 2009).

## Glucose

Glucose is the most common energy source for mammalian cells; it can be converted to pyruvate through glycolysis and produced via gluconeogenesis. Glucose uptake and usage is markedly increased in cancer cells (Bao *et al*., 2019; Ishikawa *et al*., 1993; Warburg, 1925; Warburg, 1956). Several studies indicate that elevated glucose promotes GBM cell growth, and hyperglycemia is a condition associated with worse outcomes (Bao *et al*., 2019; Derr *et al*., 2009; Ishikawa *et al*., 1993; Tieu *et al*., 2015).

## Lactate

Lactate (2-hydroxypropanoic acid) is produced by lactate dehydrogenase-A (LDH-A) in hypoxic conditions. Despite ample oxygen levels, cancer cells preferentially produce lactate and rely on substrate level phosphorylation for their energy (Seyfried and Shelton, 2010). Studies have shown that lactate has multifactorial roles in tumorigenesis, functioning as a signaling molecule for tumor angiogenesis and contributing to tumor inflammation and growth by attracting macrophages that then secrete cytokines such as IL-23/IL-17 and growth factors (Shime *et al*., 2008; Sonveaux *et al*., 2012; Végran *et al*., 2011). Amongst patients with high grade gliomas undergoing tumor resection, pretreatment serum lactate was found to be a strong prognostic biomarker (Mariappan *et al*., 2015; Shih *et al*., 2017). Overexpression of LDH-A has also been reported in GBM cell lines and is associated with increased glycolysis, growth, immune evasion, and decreased cell death (Kahlon *et al*., 2016; Li *et al*., 2016).

## Isocitrate Dehydrogenase, D-2-hydroxyglutarate

IDH catalyzes the oxidative decarboxylation of isocitrate to α-KG with the reduction of NADP+ to NADPH. IDH1 and 2 are homologous enzymes that differ in that IDH1 is localized to the cytosol while IDH2 resides in the mitochondrial matrix.

IDH1 mutations at R132 have been associated with astrocytomas, GBM, and oligodendrogliomas, and IDH2 mutations at R172 have been linked with gliomas (Dang *et al*., 2010; Yan *et al*., 2009).IDH1/2 have been linked to enhanced reductive carboxylation in hypoxia for de novo lipogenesis (Metallo *et al*., 2011). In a study of IDH1-mutant (IDH1-mt) gliomas, one study found decreased triglycerides and sphingolipids and elevated pyruvate entering the TCA cycle in IDH1-mt gliomas compared to IDH-wt (Zhou *et al*., 2019). As a prognostic marker, several studies have found that IDH1/2 mutations are linked to prolonged patient survival and therapeutic response amongst patients with gliomas and GBM (Cairncross *et al*., 2014; Chen *et al*., 2016; Parsons *et al*., 2008; Yan *et al*., 2009).

While it is well established that many patients with glioma experience at least one seizure in the course of their illness (Moots *et al*., 1995; van Breemen *et al*., 2007), epileptogenicity remains poorly understood. Recent studies have pointed to IDH1/2-mt cancers as vulnerable to higher seizure incidence relative to IDH-wt (Chen *et al*., 2017; Zhong *et al*., 2015). This may be due to the production of D-2-hydroxyglutarate (D2HG) via NADPH-dependent reduction of α-KG in IDH1/2-mt cancers (Dang *et al*., 2010; Lee *et al*., 2019). α-KG is believed to have anti-epileptic properties (Yamamoto and Mohanan, 2003) and D2HG competitively inhibits α-KG dependent enzymes (Xu *et al*., 2011) which may ultimately contribute to carcinogenesis and seizures. Additionally, D2HG structurally resembles the excitatory neurotransmitter, glutamate, and D2HG in IDH1-mt gliomas have been associated with seizure incidence due to their agonistic actionon the NMDA receptor (Chen *et al*., 2017).

## Glutamate

Glutamate is a CNS excitatory neurotransmitter. Studies indicate that gliomas release glutamate and this has been implicated in tumor expansion (Takano *et al*., 2001), cellular edema (Savaskan *et al*., 2008), and tumor associated excitotoxicity and epilepsy (Buckingham and Robel, 2013; de Groot and Sontheimer, 2011; Yuen *et al*., 2012). Interestingly, Nakae *et al*. found that while higher glutamate concentration was in IDH-wt glioma relative to IDH-mt, glutamate was not associated with high preoperative seizure frequency (Nakae *et al*., 2021). Rather, levels of total N-acetyl-L-aspartate were found to be statistically correlated and were independent predicators of preoperative seizures (Nakae *et al*., 2021).

Glutamate can be derived from glutamine, the most abundant amino acid in the body. Glutamine transports nitrogen for purine, pyrimidine, and fatty acid biosynthesis and can serve as an agent to fuel the TCA cycle. Conversely, glutamine can be generated from glutamate by a via the glutamine-glutamate cycle (Bak *et al*., 2006). In summary, glutamate is transported to astrocytes where it is converted to glutamine by glutamine synthetase. The newly synthesized glutamine then arrives at presynaptic neurons where it is hydrolyzed to glutamate by glutaminase, packaged into synaptic vesicles and released into the synaptic cleft during neurotransmission. The phrase “glutamine addiction” is used to describe cancer cells’ exorbitant consumption of glutamine as an energy source and it has been posited that glutamine may be a key marker for glioma progression (Ekici *et al*., 2020; Márquez *et al*., 2017; Wise and Thompson, 2010).

## Additional Metabolites

Advancements in metabolomics have directed attention at other metabolites and LMWMs that can serve as glioma biomarkers and therapeutic targets, e.g. proline, arginine, methionine, kynurenate, and tryptophan. Proline is oxidized to glutamate in the mitochondria and has been linked to glutamate metabolism in GBM and cell proliferation (Cappelletti *et al*., 2018). In a recent systematic review, Sawicka *et al*. found proline to be a prognostic factor and a signal of malignancy (Sawicka *et al*., 2022).

There are also GBM studies that indicate theprognostic potential of arginine, methionine, kynurenate, and kynurenine (Palanichamy *et al*., 2016; Shen *et al*., 2018). Kynurenine is a metabolite of tryptophan and is involved in immune evasion in GBM and thus tumor progression and poor prognosis. Overall, metabolomic studies have illuminated how metabolites show promise to be reliable prognosticators (Erb *et al*., 2008).

## Lipids

Though lipidomics is an individual area of study dedicated to identifying and understanding cellular lipids and their pathways, it is worthwhile to discuss key alterations that overlap with metabolomics. Lipids serve as significant infrastructure components across cells and energy sources. For various cancer types, critical lipid metabolism regulators including sterol regulatory element-binding proteins (SREBPs), acetyl-CoA carboxylase, fatty acid synthase, and stearoyl-CoA desaturase are up-regulated and extensively covered in another review (Cheng *et al*., 2018). In glioblastomas, key lipid components that induce cancerous growth and spread include SREBP, sterol-O-acyltransfersase (SOAT) and lipid droplets (LDs) (Geng *et al*., 2016; Yuan *et al*., 2021). SOAT converts endoplasmic reticulum cholesterol to cholesterol esters (CE) and sequesters CE into LDs to prevent over accumulation of cholesterol and lipotoxicity. Inhibition of SOAT in xenograft models has been shown to inhibit SREBP and thereby reduce GBM growth (Geng *et al*., 2016). A separate study with a focus on LDs has shown their role in lipid metabolism exploitation by glial and GBM cells (Yuan *et al*., 2021).

Not only has lipogenesis been underscored as a method of GBM progression, but acyl carnitines presence in GBM suggests upregulation of fatty oxidation for GB cellular respiration and proliferation (Lin *et al*., 2017). In IDH1-mt gliomas, lower levels of fatty acyl chains, triglycerides and sphingolipids are posited to be due to low acyl-CoA synthetase 1which has conferred better survival outcomes (Zhou *et al*., 2019).

## Treatment

Standard of care treatment for gliomas typically consists of surgical resection and adjuvant chemotherapy and radiotherapy (Stupp *et al*., 2005), which can lead to treatment-related glioma hypermutation. However, hypermutation has been found highest in early tumorigenesis (Barthel *et al*., 2019). Recurrent IDH-wt GBM samples from patients who had received chemoradiotherapy and temozolomide (TMZ) were more likely to contain DNA methylation genes, mutations in DNA damage response genes and hypermutated phenotypes due to mutations in mismatch repair genes (Draaisma *et al*., 2020). These findings further emphasize the necessity of targeted therapeutic approaches for gliomas.

Experimental therapeutic interventions for GBM may involve a combinatorial approach. One such experimental combination approach is fluoxetine (Prozac) and TMZ. TMZ is an alkylating agent which has long been used as a chemotherapeutic agent to treat high grade gliomas due to its ability to methylate guanine at the O6 position which reduces DNA repair capabilities and ultimately induces cell apoptosis (Friedman *et al*., 2000). Fluoxetine is a SSRI antidepressant capable of selectively inhibiting sphingomyelin phosphodiesterase 1 (SMPD1) and, when combined with TMZ, is reported to reduce tumors in murine models and increase survival in GBM patients (Bi *et al*., 2021).

Immunotherapy has emerged as an attractive treatment route, but its challenges are mired in the complexities of brain tumor metabolism in the tumor microenvironment (TME) (Guerra *et al*., 2020) and CNS anatomy (Engelhardt *et al*., 2017). Current immunotherapy trials exploring immune checkpoint inhibitors, vaccine therapies, viral introduction to various CNS neoplastic cell lines, and multimodal approaches have had mixed results (Mende *et al*., 2021).

Overexpression of the G-protein coupled receptor CCR5 across multiple cancer types including GBM has become a niche of neurooncological studies that examine therapeutic targets of this receptor (Lah Turnšek *et al*., 2021). CCR5 has been implicated in GBM stem cell genesis, invasion, and spread. Kranjc *et al*. offers an extensive review of the various mechanisms and components at play between CCR5 and one of its ligands, chemokine ligand 5 (CCL5) in GBM (Kranjc *et al*., 2019).

Selective HK2 inhibition has been interrogated as another metabolic target for GBM treatment (Agnihotri *et al*., 2019). In-vivo chemoradiotherapy studies have shown HK knock out as a promising target (Vartanian *et al*., 2016) and antifungals ketoconazole and posaconazole have exhibited reduced tumor metabolism and expansion in-vitro, offering evidence for their consideration in clinical trials (Agnihotri *et al*., 2019).

Natural products derived from vegetables, fruits, spices, and herbs have also been a source of interest for GBM as they are capable of influencing GBM cell cycles, apoptosis, angiogenesis, epigenetic alterations, and reducing therapeutic resistance (Abbas *et al*., 2020). Flavonoids are naturally present in vegetables and fruits, and the flavonoids apigenin, epigallocatechin, and genistein were observed to selectively activate apoptosis in GBM cells (Das *et al*., 2010). Quercetin, another extensively studied natural flavonoid in vegetables and fruits including broccoli, red onions, apples, red grapes, cherries, and berries has been found to induce apoptosis in GBM cells and increase chemoradiotherapy effects (Kim *et al*., 2021; Pozsgai *et al*., 2013). Amongst polyphenols, resveratrol, a natural polyphenol in mulberries, grapes, and peanuts, increased apoptosis and reversed TMZ resistance in T98G glioblastoma cells, suggesting that certain natural products can serve as a beneficial adjunctive therapy (Huang *et al*., 2012). A recent review by Abbas *et al*. covers their molecular mechanisms in greater detail (Abbas *et al*., 2020).

## Resistance

While targeted therapeutics approaches offer benefit to cancer patients outside the CNS based on their tumor’s molecular characteristics, over two decades of data demonstrate that this overall approach suffers from several weaknesses in high grade glioma treatment. Most prominent among these is the propensity of subpopulations of heterogeneous tumor cells to subvert drugs that act via apoptosis, by far the most prevalent cell death mechanism engaged by currently employed therapeutic agents, leading to therapeutic resistance and poor patient outcome.

Two models theorize cancer cell heterogeneity and its consequences: clonal evolution and the cancer stem cell (CSC) hypothesis. The clonal evolution theory, proposed by Peter Nowell in 1976, suggests that the accumulation of cellular mutations via heritable and epigenetic changes generate clonal outgrowths that thrive in response to microenvironmental selection pressures (Nowell, 1976). In effect, the model resembles the Darwinian model of evolution. The application of this theory in GBM cells has been observed in a study by Wang *et al*., who found increased mutation in recurrent GBM that had been treated with standard therapy including TMZ, indicating that resistant clones survive and hypermutatewhen confronted with drug-induced pressure (Yuzawa *et al*., 2016).

The cancer stem cell (CSC) hypothesis first manifested in brain tumors in 2004, when Singh *et al*. found a subset of GBM stem cells (GSC) that were capable of initiating tumor growth via CD133+ protein surface markers (Singh *et al*., 2004). GSCs have since been observed to possess chemoresistance (Eramo *et al*., 2006) and human mesenchymal stem cells (hMSCs) inside glioma-associated hMSCs have been linked to dismal outcomes (Shahar *et al*., 2017). Chemoresistance is in part due to the increased expression of tumor growth factors such as VEGF and stromal derived factor 1 (SDF-1) which promote angiogenesis and appear to provide resistance against standard therapies such as radiation (Folkins *et al*., 2009; Harmey and Bouchier-Hayes, 2002). CSCs are also able to prevent cytotoxicity through high drug efflux by ATP-binding cassette (ABC) transporters, which are ATP-dependent membrane-bound proteins (Vasiliou *et al*., 2009). Overexpression of ABCB1, ABCB5, and ABCA13 (three ABC transporters) were found to increase TMZ resistance (Chou *et al*., 2012; Dréan *et al*., 2018; Lee *et al*., 2020).

In addition to clonal evolution and stem cell capacity to shape shift and evade cell death with TMZ, another response from GBM cells upon TMZ administration is over expression of the DNA repair protein O-6-methylguanine-DNA methyltransferase (MGMT), which confers resistance and shorter survival (Perry *et al*., 2017). Therapeutic resistance has also been linked to microRNA (miRNA) dysregulation. Shi *et al*. found that U87MG GBM cells developed resistance to TMZ by upregulation of miRNA-21 which decreased Bax/Bcl-2 and caspase-3 activity (Shi *et al*., 2010).

## Conclusion

Despite scientific advancements made in cancer research, high grade gliomas remain enigmatic. Just as the discovery of IDH mutation sparked great interest in cancer metabolism in the neuro-oncology field, we have the opportunity to take it to the next step using metabolomics to help understand disease progression and changes in cancer profiles across all cancer types (Crooks *et al*., 2021; Kwon *et al*., 2015). Our summary of metabolomic studies that pertain to high grade gliomas, spanning metabolic pathways, metabolites, and therapeutic interventions underscores the significance of metabolomics as a way to better understand and treat high grade gliomas.

## Abbreviations:

WHO: World Health Organization
HGG: high grade glioma
LMWM: low molecular weight molecule
α-KG: α-ketoglutarate
GBM: glioblastoma
IDH: isocitrate dehydrogenase
IDH-mt: IDH-mutant
IDH-wt: IDH-wild type
TCA: tricarboxylic acid cycle
TMZ: temozolomide
SMPD1: sphingomyelin phosphodiesterase 1
G6P: glucose-6-phosphate
LDH-A: lactate dehydrogenase-A
D2HG: D-2-hydroxyglutarat
SREBPs: Sterol regulatory element-binding proteins
SOAT: sterol-O-acyltransfersase
LDs: lipid droplets
CD: cholesterol ester
CNS: central nervous system
TME: tumor microenvironment
CCL5: chemokine ligand 5
MGMT: O-6-methylguanine-DNA methyltransferas
CSC: cancer stem cel
hMSC: human mesenchymal cel
ABC transporter: ATP-binding cassette transporte
HK2: hexokinase 2
SDH: succinate dehydrogenase
FH: fumarate hydratase
miRNA: microRNA
HIF-1a: hypoxia-inducible factor-1a

## References

[R1] Abbas MN, Kausar S and Cui H (2020) Therapeutic potential of natural products in glioblastoma treatment: targeting key glioblastoma signaling pathways and epigenetic alterations. Clin Transl Oncol 22, 963–977. 31630356 10.1007/s12094-019-02227-3

[R2] Agnihotri S, Mansouri S, Burrell K, Li M, Mamatjan Y, Liu J, Nejad R, Kumar S, Jalali S, Singh SK, Vartanian A, Chen EX, Karimi S, Singh O, Bunda S, Mansouri A, Aldape KD and Zadeh G (2019) Ketoconazole and Posaconazole Selectively Target HK2-expressing Glioblastoma Cells. Clin Cancer Res 25, 844–855. 30322879 10.1158/1078-0432.CCR-18-1854 PMC8103287

[R3] Bak LK, Schousboe A and Waagepetersen HS (2006) The glutamate/GABA-glutamine cycle: aspects of transport, neurotransmitter homeostasis and ammonia transfer. J Neurochem 98, 641–53. 16787421 10.1111/j.1471-4159.2006.03913.x

[R4] Bao Z, Chen K, Krepel S, Tang P, Gong W, Zhang M, Liang W, Trivett A, Zhou M and Wang JM (2019) High Glucose Promotes Human Glioblastoma Cell Growth by Increasing the Expression and Function of Chemoattractant and Growth Factor Receptors. Transl Oncol 12, 1155–1163. 31207546 10.1016/j.tranon.2019.04.016 PMC6580091

[R5] Barthel FP, Johnson KC, Varn FS, Moskalik AD, Tanner G, Kocakavuk E, Anderson KJ, Abiola O, Aldape K, Alfaro KD, Alpar D, Amin SB, Ashley DM, Bandopadhayay P, Barnholtz-Sloan JS, Beroukhim R, Bock C, Brastianos PK, Brat DJ, Brodbelt AR, Bruns AF, Bulsara KR, Chakrabarty A, Chakravarti A, Chuang JH, Claus EB, Cochran EJ, Connelly J, Costello JF, Finocchiaro G, Fletcher MN, French PJ, Gan HK, Gilbert MR, Gould PV, Grimmer MR, Iavarone A, Ismail A, Jenkinson MD, Khasraw M, Kim H, Kouwenhoven MCM, LaViolette PS, Li M, Lichter P, Ligon KL, Lowman AK, Malta TM, Mazor T, McDonald KL, Molinaro AM, Nam DH, Nayyar N, Ng HK, Ngan CY, Niclou SP, Niers JM, Noushmehr H, Noorbakhsh J, Ormond DR, Park CK, Poisson LM, Rabadan R, Radlwimmer B, Rao G, Reifenberger G, Sa JK, Schuster M, Shaw BL, Short SC, Smitt PAS, Sloan AE, Smits M, Suzuki H, Tabatabai G, Van Meir EG, Watts C, Weller M, Wesseling P, Westerman BA, Widhalm G, Woehrer A, Yung WKA, Zadeh G, Huse JT, De Groot JF, Stead LF, Verhaak RGW and Consortium G (2019) Longitudinal molecular trajectories of diffuse glioma in adults. Nature 576, 112–120. 31748746 10.1038/s41586-019-1775-1 PMC6897368

[R6] Bi J, Khan A, Tang J, Armando AM, Wu S, Zhang W, Gimple RC, Reed A, Jing H, Koga T, Wong IT, Gu Y, Miki S, Yang H, Prager B, Curtis EJ, Wainwright DA, Furnari FB, Rich JN, Cloughesy TF, Kornblum HI, Quehenberger O, Rzhetsky A, Cravatt BF and Mischel PS (2021) Targeting glioblastoma signaling and metabolism with a re-purposed brain-penetrant drug. Cell Rep 37, 109957. 34731610 10.1016/j.celrep.2021.109957 PMC8856626

[R7] Buckingham SC and Robel S (2013) Glutamate and tumor-associated epilepsy: glial cell dysfunction in the peritumoral environment. Neurochem Int 63, 696–701. 23385090 10.1016/j.neuint.2013.01.027 PMC3664257

[R8] Cairncross JG, Wang M, Jenkins RB, Shaw EG, Giannini C, Brachman DG, Buckner JC, Fink KL, Souhami L, Laperriere NJ, Huse JT, Mehta MP and Curran WJ Jr. (2014) Benefit from procarbazine, lomustine, and vincristine in oligodendroglial tumors is associated with mutation of IDH. J Clin Oncol 32, 783–90. 24516018 10.1200/JCO.2013.49.3726 PMC3940537

[R9] Cappelletti P, Tallarita E, Rabattoni V, Campomenosi P, Sacchi S and Pollegioni L (2018) Proline oxidase controls proline, glutamate, and glutamine cellular concentrations in a U87 glioblastoma cell line. PLoS One 13, e0196283. 29694413 10.1371/journal.pone.0196283 PMC5918996

[R10] Chakravarti A, Zhai G, Suzuki Y, Sarkesh S, Black PM, Muzikansky A and Loeffler JS (2004) The prognostic significance of phosphatidylinositol 3-kinase pathway activation in human gliomas. J Clin Oncol 22, 1926–33. 15143086 10.1200/JCO.2004.07.193

[R11] Chen H, Judkins J, Thomas C, Wu M, Khoury L, Benjamin CG, Pacione D, Golfinos JG, Kumthekar P, Ghamsari F, Chen L, Lein P, Chetkovich DM, Snuderl M and Horbinski C (2017) Mutant IDH1 and seizures in patients with glioma. Neurology 88, 1805–1813. 28404805 10.1212/WNL.0000000000003911 PMC5419985

[R12] Chen JQ and Russo J (2012) Dysregulation of glucose transport, glycolysis, TCA cycle and glutaminolysis by oncogenes and tumor suppressors in cancer cells. Biochim Biophys Acta 1826, 370–84. 22750268 10.1016/j.bbcan.2012.06.004

[R13] Chen JR, Yao Y, Xu HZ and Qin ZY (2016) Isocitrate Dehydrogenase (IDH)1/2 Mutations as Prognostic Markers in Patients With Glioblastomas. Medicine (Baltimore) 95, e2583. 26945349 10.1097/MD.0000000000002583 PMC4782833

[R14] Cheng C, Geng F, Cheng X and Guo D (2018) Lipid metabolism reprogramming and its potential targets in cancer. Cancer Commun (Lond) 38, 27. 29784041 10.1186/s40880-018-0301-4 PMC5993136

[R15] Choe G, Horvath S, Cloughesy TF, Crosby K, Seligson D, Palotie A, Inge L, Smith BL, Sawyers CL and Mischel PS (2003) Analysis of the phosphatidylinositol 3’-kinase signaling pathway in glioblastoma patients in vivo. Cancer Res 63, 2742–6. 12782577

[R16] Chou CW, Wang CC, Wu CP, Lin YJ, Lee YC, Cheng YW and Hsieh CH (2012) Tumor cycling hypoxia induces chemoresistance in glioblastoma multiforme by upregulating the expression and function of ABCB1. Neuro Oncol 14, 1227–38. 22946104 10.1093/neuonc/nos195 PMC3452342

[R17] Crooks DR, Maio N, Lang M, Ricketts CJ, Vocke CD, Gurram S, Turan S, Kim YY, Cawthon GM, Sohelian F, De Val N, Pfeiffer RM, Jailwala P, Tandon M, Tran B, Fan TW, Lane AN, Ried T, Wangsa D, Malayeri AA, Merino MJ, Yang Y, Meier JL, Ball MW, Rouault TA, Srinivasan R and Linehan WM (2021) Mitochondrial DNA alterations underlie an irreversible shift to aerobic glycolysis in fumarate hydratase-deficient renal cancer. Sci Signal 14. 10.1126/scisignal.abc4436 PMC8039187 33402335

[R18] Dang L, White DW, Gross S, Bennett BD, Bittinger MA, Driggers EM, Fantin VR, Jang HG, Jin S, Keenan MC, Marks KM, Prins RM, Ward PS, Yen KE, Liau LM, Rabinowitz JD, Cantley LC, Thompson CB, Vander Heiden MG and Su SM (2010) Cancer-associated IDH1 mutations produce 2-hydroxyglutarate. Nature 465, 966. 20559394 10.1038/nature09132 PMC3766976

[R19] Das A, Banik NL and Ray SK (2010) Flavonoids activated caspases for apoptosis in human glioblastoma T98G and U87MG cells but not in human normal astrocytes. Cancer 116, 164–76. 19894226 10.1002/cncr.24699 PMC3159962

[R20] de Groot J and Sontheimer H (2011) Glutamate and the biology of gliomas. Glia 59, 1181–9. 21192095 10.1002/glia.21113 PMC3107875

[R21] Derr RL, Ye X, Islas MU, Desideri S, Saudek CD and Grossman SA (2009) Association between hyperglycemia and survival in patients with newly diagnosed glioblastoma. J Clin Oncol 27, 1082–6. 19139429 10.1200/JCO.2008.19.1098 PMC2667812

[R22] Draaisma K, Chatzipli A, Taphoorn M, Kerkhof M, Weyerbrock A, Sanson M, Hoeben A, Lukacova S, Lombardi G, Leenstra S, Hanse M, Fleischeuer R, Watts C, McAbee J, Angelopoulos N, Gorlia T, Golfinopoulos V, Kros JM, Verhaak RGW, Bours V, van den Bent MJ, McDermott U, Robe PA and French PJ (2020) Molecular Evolution of IDH Wild-Type Glioblastomas Treated With Standard of Care Affects Survival and Design of Precision Medicine Trials: A Report From the EORTC 1542 Study. J Clin Oncol 38, 81–99. 31743054 10.1200/JCO.19.00367

[R23] Dréan A, Rosenberg S, Lejeune FX, Goli L, Nadaradjane AA, Guehennec J, Schmitt C, Verreault M, Bielle F, Mokhtari K, Sanson M, Carpentier A, Delattre JY and Idbaih A (2018) ATP binding cassette (ABC) transporters: expression and clinical value in glioblastoma. J Neurooncol 138, 479–486. 29520610 10.1007/s11060-018-2819-3

[R24] Eagle H (1955) The minimum vitamin requirements of the L and HeLa cells in tissue culture, the production of specific vitamin deficiencies, and their cure. J Exp Med 102, 595–600. 13271674 10.1084/jem.102.5.595 PMC2136536

[R25] Ekici S, Risk BB, Neill SG, Shu HK and Fleischer CC (2020) Characterization of dysregulated glutamine metabolism in human glioma tissue with (1)H NMR. Sci Rep 10, 20435. 33235296 10.1038/s41598-020-76982-7 PMC7686482

[R26] Eng C, Kiuru M, Fernandez MJ and Aaltonen LA (2003) A role for mitochondrial enzymes in inherited neoplasia and beyond. Nat Rev Cancer 3, 193–202. 12612654 10.1038/nrc1013

[R27] Engelhardt B, Vajkoczy P and Weller RO (2017) The movers and shapers in immune privilege of the CNS. Nat Immunol 18, 123–131. 28092374 10.1038/ni.3666

[R28] Eramo A, Ricci-Vitiani L, Zeuner A, Pallini R, Lotti F, Sette G, Pilozzi E, Larocca LM, Peschle C and De Maria R (2006) Chemotherapy resistance of glioblastoma stem cells. Cell Death Differ 13, 1238–41. 16456578 10.1038/sj.cdd.4401872

[R29] Erb G, Elbayed K, Piotto M, Raya J, Neuville A, Mohr M, Maitrot D, Kehrli P and Namer IJ (2008) Toward improved grading of malignancy in oligodendrogliomas using metabolomics. Magn Reson Med 59, 959–65. 18429037 10.1002/mrm.21486

[R30] Fischer I, Gagner JP, Law M, Newcomb EW and Zagzag D (2005) Angiogenesis in gliomas: biology and molecular pathophysiology. Brain Pathol 15, 297–310. 16389942 10.1111/j.1750-3639.2005.tb00115.x PMC8096031

[R31] Folkins C, Shaked Y, Man S, Tang T, Lee CR, Zhu Z, Hoffman RM and Kerbel RS (2009) Glioma tumor stem-like cells promote tumor angiogenesis and vasculogenesis via vascular endothelial growth factor and stromal-derived factor 1. Cancer Res 69, 7243–51. 19738068 10.1158/0008-5472.CAN-09-0167 PMC3409689

[R32] Friedman HS, Kerby T and Calvert H (2000) Temozolomide and treatment of malignant glioma. Clin Cancer Res 6, 2585–97. 10914698

[R33] Geng F, Cheng X, Wu X, Yoo JY, Cheng C, Guo JY, Mo X, Ru P, Hurwitz B, Kim SH, Otero J, Puduvalli V, Lefai E, Ma J, Nakano I, Horbinski C, Kaur B, Chakravarti A and Guo D (2016) Inhibition of SOAT1 Suppresses Glioblastoma Growth via Blocking SREBP-1-Mediated Lipogenesis. Clin Cancer Res 22, 5337–5348. 27281560 10.1158/1078-0432.CCR-15-2973 PMC5093025

[R34] Guerra L, Bonetti L and Brenner D (2020) Metabolic Modulation of Immunity: A New Concept in Cancer Immunotherapy. Cell Rep 32, 107848. 32640218 10.1016/j.celrep.2020.107848

[R35] Harmey JH and Bouchier-Hayes D (2002) Vascular endothelial growth factor (VEGF), a survival factor for tumour cells: implications for anti-angiogenic therapy. Bioessays 24, 280–3. 11891765 10.1002/bies.10043

[R36] Huang H, Lin H, Zhang X and Li J (2012) Resveratrol reverses temozolomide resistance by downregulation of MGMT in T98G glioblastoma cells by the NF-κB-dependent pathway. Oncol Rep 27, 2050–6. 22426504 10.3892/or.2012.1715

[R37] Ishikawa M, Kikuchi H, Miyatake S, Oda Y, Yonekura Y and Nishizawa S (1993) Glucose consumption in recurrent gliomas. Neurosurgery 33, 28–33. 8355844 10.1227/00006123-199307000-00004

[R38] Jin L and Zhou Y (2019) Crucial role of the pentose phosphate pathway in malignant tumors. Oncol Lett 17, 4213–4221. 30944616 10.3892/ol.2019.10112 PMC6444344

[R39] Kahlon AS, Alexander M, Kahlon A and Wright J (2016) Lactate levels with glioblastoma multiforme. Proc (Bayl Univ Med Cent) 29, 313–4. 27365883 10.1080/08998280.2016.11929449 PMC4900781

[R40] Khan TA, Loftus TJ, Filiberto AC, Ozrazgat-Baslanti T, Ruppert MM, Bandyopadhyay S, Laiakis EC, Arnaoutakis DJ and Bihorac A (2021) Metabolomic Profiling for Diagnosis and Prognostication in Surgery: A Scoping Review. Ann Surg 273, 258–268. 32482979 10.1097/SLA.0000000000003935 PMC7704904

[R41] Kim HI, Lee SJ, Choi YJ, Kim MJ, Kim TY and Ko SG (2021) Quercetin Induces Apoptosis in Glioblastoma Cells by Suppressing Axl/IL-6/STAT3 Signaling Pathway. Am J Chin Med 49, 767–784. 33657989 10.1142/S0192415X21500361

[R42] Kranjc MK, Novak M, Pestell RG and Lah TT (2019) Cytokine CCL5 and receptor CCR5 axis in glioblastoma multiforme. Radiol Oncol 53, 397–406. 31747383 10.2478/raon-2019-0057 PMC6884928

[R43] Kwon H, Oh S, Jin X, An YJ and Park S (2015) Cancer metabolomics in basic science perspective. Arch Pharm Res 38, 372–80. 25630795 10.1007/s12272-015-0552-4

[R44] Lah Turnšek T, Jiao X, Novak M, Jammula S, Cicero G, Ashton AW, Joyce D and Pestell RG (2021) An Update on Glioblastoma Biology, Genetics, and Current Therapies: Novel Inhibitors of the G Protein-Coupled Receptor CCR5. Int J Mol Sci 22. 10.3390/ijms22094464 PMC8123168 33923334

[R45] Lee CAA, Banerjee P, Wilson BJ, Wu S, Guo Q, Berg G, Karpova S, Mishra A, Lian JW, Tran J, Emmerich M, Murphy GF, Frank MH and Frank NY (2020) Targeting the ABC transporter ABCB5 sensitizes glioblastoma to temozolomide-induced apoptosis through a cell-cycle checkpoint regulation mechanism. J Biol Chem 295, 7774–7788. 32317280 10.1074/jbc.RA120.013778 PMC7261782

[R46] Lee JE, Jeun SS, Kim SH, Yoo CY, Baek HM and Yang SH (2019) Metabolic profiling of human gliomas assessed with NMR. J Clin Neurosci 68, 275–280. 31409545 10.1016/j.jocn.2019.07.078

[R47] Li J, Zhu S, Tong J, Hao H, Yang J, Liu Z and Wang Y (2016) Suppression of lactate dehydrogenase A compromises tumor progression by downregulation of the Warburg effect in glioblastoma. Neuroreport 27, 110–5. 26694942 10.1097/WNR.0000000000000506 PMC4712768

[R48] Lin H, Patel S, Affleck VS, Wilson I, Turnbull DM, Joshi AR, Maxwell R and Stoll EA (2017) Fatty acid oxidation is required for the respiration and proliferation of malignant glioma cells. Neuro Oncol 19, 43–54. 27365097 10.1093/neuonc/now128 PMC5193020

[R49] Louis DN, Perry A, Wesseling P, Brat DJ, Cree IA, Figarella-Branger D, Hawkins C, Ng HK, Pfister SM, Reifenberger G, Soffietti R, von Deimling A and Ellison DW (2021) The 2021 WHO Classification of Tumors of the Central Nervous System: a summary. Neuro Oncol 23, 1231–1251. 34185076 10.1093/neuonc/noab106 PMC8328013

[R50] Mariappan R, Venkatraghavan L, Vertanian A, Agnihotri S, Cynthia S, Reyhani S, Tung T, Khan OH and Zadeh G (2015) Serum lactate as a potential biomarker of malignancy in primary adult brain tumours. J Clin Neurosci 22, 144–8. 25172017 10.1016/j.jocn.2014.06.005

[R51] Márquez J, Alonso FJ, Matés JM, Segura JA, Martín-Rufián M and Campos-Sandoval JA (2017) Glutamine Addiction In Gliomas. Neurochem Res 42, 1735–1746. 28281102 10.1007/s11064-017-2212-1

[R52] Mende AL, Schulte JD, Okada H and Clarke JL (2021) Current Advances in Immunotherapy for Glioblastoma. Curr Oncol Rep 23, 21. 33496872 10.1007/s11912-020-01007-5 PMC7838142

[R53] Metallo CM, Gameiro PA, Bell EL, Mattaini KR, Yang J, Hiller K, Jewell CM, Johnson ZR, Irvine DJ, Guarente L, Kelleher JK, Vander Heiden MG, Iliopoulos O and Stephanopoulos G (2011) Reductive glutamine metabolism by IDH1 mediates lipogenesis under hypoxia. Nature 481, 380–4. 22101433 10.1038/nature10602 PMC3710581

[R54] Moots PL, Maciunas RJ, Eisert DR, Parker RA, Laporte K and Abou-Khalil B (1995) The course of seizure disorders in patients with malignant gliomas. Arch Neurol 52, 717–24. 7619029 10.1001/archneur.1995.00540310091021

[R55] Nakae S, Kumon M, Murayama K, Ohba S, Sasaki H, Inamasu J, Kuwahara K, Yamada S, Abe M and Hirose Y (2021) Association of preoperative seizures with tumor metabolites quantified by magnetic resonance spectroscopy in gliomas. Sci Rep 11, 7927. 33846339 10.1038/s41598-021-86487-6 PMC8041994

[R56] Nicholson JK and Lindon JC (2008) Systems biology: Metabonomics. Nature 455, 1054–6. 18948945 10.1038/4551054a

[R57] Nowell PC (1976) The clonal evolution of tumor cell populations. Science 194, 23–8. 959840 10.1126/science.959840

[R58] Palanichamy K, Thirumoorthy K, Kanji S, Gordon N, Singh R, Jacob JR, Sebastian N, Litzenberg KT, Patel D, Bassett E, Ramasubramanian B, Lautenschlaeger T, Fischer SM, Ray-Chaudhury A and Chakravarti A (2016) Methionine and Kynurenine Activate Oncogenic Kinases in Glioblastoma, and Methionine Deprivation Compromises Proliferation. Clin Cancer Res 22, 3513–23. 26936918 10.1158/1078-0432.CCR-15-2308 PMC4947420

[R59] Parsons DW, Jones S, Zhang X, Lin JC, Leary RJ, Angenendt P, Mankoo P, Carter H, Siu IM, Gallia GL, Olivi A, McLendon R, Rasheed BA, Keir S, Nikolskaya T, Nikolsky Y, Busam DA, Tekleab H, Diaz LA Jr., Hartigan J, Smith DR, Strausberg RL, Marie SK, Shinjo SM, Yan H, Riggins GJ, Bigner DD, Karchin R, Papadopoulos N, Parmigiani G, Vogelstein B, Velculescu VE and Kinzler KW (2008) An integrated genomic analysis of human glioblastoma multiforme. Science 321, 1807–12. 18772396 10.1126/science.1164382 PMC2820389

[R60] Patra KC and Hay N (2014) The pentose phosphate pathway and cancer. Trends Biochem Sci 39, 347–54. 25037503 10.1016/j.tibs.2014.06.005 PMC4329227

[R61] Patti GJ, Yanes O and Siuzdak G (2012) Innovation: Metabolomics: the apogee of the omics trilogy. Nat Rev Mol Cell Bio l13, 263–9. 10.1038/nrm3314 PMC3682684 22436749

[R62] Pavlova NN and Thompson CB (2016) The Emerging Hallmarks of Cancer Metabolism. Cell Metab 23, 27–47. 26771115 10.1016/j.cmet.2015.12.006 PMC4715268

[R63] Perry JR, Laperriere N, O’Callaghan CJ, Brandes AA, Menten J, Phillips C, Fay M, Nishikawa R, Cairncross JG, Roa W, Osoba D, Rossiter JP, Sahgal A, Hirte H, Laigle-Donadey F, Franceschi E, Chinot O, Golfinopoulos V, Fariselli L, Wick A, Feuvret L, Back M, Tills M, Winch C, Baumert BG, Wick W, Ding K and Mason WP (2017) Short-Course Radiation plus Temozolomide in Elderly Patients with Glioblastoma. N Engl J Med 376, 1027–1037. 28296618 10.1056/NEJMoa1611977

[R64] Pozsgai E, Bellyei S, Cseh A, Boronkai A, Racz B, Szabo A, Sumegi B and Hocsak E (2013) Quercetin increases the efficacy of glioblastoma treatment compared to standard chemoradiotherapy by the suppression of PI-3-kinase-Akt pathway. Nutr Cancer 65, 1059–66. 24032376 10.1080/01635581.2013.810291

[R65] Savaskan NE, Heckel A, Hahnen E, Engelhorn T, Doerfler A, Ganslandt O, Nimsky C, Buchfelder M and Eyüpoglu IY (2008) Small interfering RNA-mediated xCT silencing in gliomas inhibits neurodegeneration and alleviates brain edema. Nat Med 14, 629–32. 18469825 10.1038/nm1772

[R66] Sawicka MM, Sawicki K, Łysoń T, Polityńska B and Miltyk W (2022) Proline Metabolism in Malignant Gliomas: A Systematic Literature Review. Cancers (Basel) 14. 10.3390/cancers14082030 PMC9027994 35454935

[R67] Seyfried TN and Shelton LM (2010) Cancer as a metabolic disease. Nutr Metab (Lond) 7, 7. 20181022 10.1186/1743-7075-7-7 PMC2845135

[R68] Shahar T, Rozovski U, Hess KR, Hossain A, Gumin J, Gao F, Fuller GN, Goodman L, Sulman EP and Lang FF (2017) Percentage of mesenchymal stem cells in high-grade glioma tumor samples correlates with patient survival. Neuro Oncol 19, 660–668. 28453745 10.1093/neuonc/now239 PMC5464439

[R69] Shen J, Song R, Hodges TR, Heimberger AB and Zhao H (2018) Identification of metabolites in plasma for predicting survival in glioblastoma. Mol Carcinog 57, 1078–1084. 29603794 10.1002/mc.22815

[R70] Shi L, Chen J, Yang J, Pan T, Zhang S and Wang Z (2010) MiR-21 protected human glioblastoma U87MG cells from chemotherapeutic drug temozolomide induced apoptosis by decreasing Bax/Bcl-2 ratio and caspase-3 activity. Brain Res 1352, 255–64. 20633539 10.1016/j.brainres.2010.07.009

[R71] Shih CC, Lee TS, Tsuang FY, Lin PL, Cheng YJ, Cheng HL and Wu CY (2017) Pretreatment serum lactate level as a prognostic biomarker in patients undergoing supratentorial primary brain tumor resection. Oncotarget 8, 63715–63723. 28969023 10.18632/oncotarget.18891 PMC5609955

[R72] Shime H, Yabu M, Akazawa T, Kodama K, Matsumoto M, Seya T and Inoue N (2008) Tumor-secreted lactic acid promotes IL-23/IL-17 proinflammatory pathway. J Immunol 180, 7175–83. 18490716 10.4049/jimmunol.180.11.7175

[R73] Singh SK, Hawkins C, Clarke ID, Squire JA, Bayani J, Hide T, Henkelman RM, Cusimano MD and Dirks PB (2004) Identification of human brain tumour initiating cells. Nature 432, 396–401. 15549107 10.1038/nature03128

[R74] Sonveaux P, Copetti T, De Saedeleer CJ, Végran F, Verrax J, Kennedy KM, Moon EJ, Dhup S, Danhier P, Frérart F, Gallez B, Ribeiro A, Michiels C, Dewhirst MW and Feron O (2012) Targeting the lactate transporter MCT1 in endothelial cells inhibits lactate-induced HIF-1 activation and tumor angiogenesis. PLoS One 7, e33418. 22428047 10.1371/journal.pone.0033418 PMC3302812

[R75] Spratlin JL, Serkova NJ and Eckhardt SG (2009) Clinical applications of metabolomics in oncology: a review. Clin Cancer Res 15, 431–40. 19147747 10.1158/1078-0432.CCR-08-1059 PMC2676437

[R76] Stupp R, Mason WP, van den Bent MJ, Weller M, Fisher B, Taphoorn MJ, Belanger K, Brandes AA, Marosi C, Bogdahn U, Curschmann J, Janzer RC, Ludwin SK, Gorlia T, Allgeier A, Lacombe D, Cairncross JG, Eisenhauer E and Mirimanoff RO (2005) Radiotherapy plus concomitant and adjuvant temozolomide for glioblastoma. N Engl J Med 352, 987–96. 15758009 10.1056/NEJMoa043330

[R77] Takano T, Lin JH, Arcuino G, Gao Q, Yang J and Nedergaard M (2001) Glutamate release promotes growth of malignant gliomas. Nat Med 7, 1010–5. 11533703 10.1038/nm0901-1010

[R78] Talley JT and Mohiuddin SS (2022) Biochemistry, Fatty Acid Oxidation, StatPearls, StatPearls Publishing Copyright © 2022, StatPearls Publishing LLC., Treasure Island (FL). 32310462

[R79] Tieu MT, Lovblom LE, McNamara MG, Mason W, Laperriere N, Millar BA, Ménard C, Kiehl TR, Perkins BA and Chung C (2015) Impact of glycemia on survival of glioblastoma patients treated with radiation and temozolomide. J Neurooncol 124, 119–26. 26015297 10.1007/s11060-015-1815-0 PMC4498235

[R80] van Breemen MS, Wilms EB and Vecht CJ (2007) Epilepsy in patients with brain tumours: epidemiology, mechanisms, and management. Lancet Neuro l6, 421–30. 10.1016/S1474-4422(07)70103-5 17434097

[R81] Vartanian A, Agnihotri S, Wilson MR, Burrell KE, Tonge PD, Alamsahebpour A, Jalali S, Taccone MS, Mansouri S, Golbourn B, Aldape KD and Zadeh G (2016) Targeting hexokinase 2 enhances response to radio-chemotherapy in glioblastoma. Oncotarget 7, 69518–69535. 27588472 10.18632/oncotarget.11680 PMC5342495

[R82] Vasiliou V, Vasiliou K and Nebert DW (2009) Human ATP-binding cassette (ABC) transporter family. Hum Genomics 3, 281–90. 19403462 10.1186/1479-7364-3-3-281 PMC2752038

[R83] Végran F, Boidot R, Michiels C, Sonveaux P and Feron O (2011) Lactate influx through the endothelial cell monocarboxylate transporter MCT1 supports an NF-κB/IL-8 pathway that drives tumor angiogenesis. Cancer Res 71, 2550–60. 21300765 10.1158/0008-5472.CAN-10-2828

[R84] Wang Z, Liu F, Fan N, Zhou C, Li D, Macvicar T, Dong Q, Bruns CJ and Zhao Y (2020) Targeting Glutaminolysis: New Perspectives to Understand Cancer Development and Novel Strategies for Potential Target Therapies. Front Oncol 10, 589508. 33194749 10.3389/fonc.2020.589508 PMC7649373

[R85] Warburg O (1925) The Metabolism of Carcinoma Cells1. The Journal of Cancer Research 9, 148–163.

[R86] Warburg O (1956) On the origin of cancer cells. Science 123, 309–14. 13298683 10.1126/science.123.3191.309

[R87] Wise DR and Thompson CB (2010) Glutamine addiction: a new therapeutic target in cancer. Trends Biochem Sci 35, 427–33. 20570523 10.1016/j.tibs.2010.05.003 PMC2917518

[R88] Wolf A, Agnihotri S, Micallef J, Mukherjee J, Sabha N, Cairns R, Hawkins C and Guha A (2011) Hexokinase 2 is a key mediator of aerobic glycolysis and promotes tumor growth in human glioblastoma multiforme. J Exp Med 208, 313–26. 21242296 10.1084/jem.20101470 PMC3039857

[R89] Xu W, Yang H, Liu Y, Yang Y, Wang P, Kim SH, Ito S, Yang C, Wang P, Xiao MT, Liu LX, Jiang WQ, Liu J, Zhang JY, Wang B, Frye S, Zhang Y, Xu YH, Lei QY, Guan KL, Zhao SM and Xiong Y (2011) Oncometabolite 2-hydroxyglutarate is a competitive inhibitor of α-ketoglutarate-dependent dioxygenases. Cancer Cell 19, 17–30. 21251613 10.1016/j.ccr.2010.12.014 PMC3229304

[R90] Yamamoto HA and Mohanan PV (2003) Effect of alpha-ketoglutarate and oxaloacetate on brain mitochondrial DNA damage and seizures induced by kainic acid in mice. Toxicol Lett 143, 115–22. 12749815 10.1016/s0378-4274(03)00114-0

[R91] Yan H, Parsons DW, Jin G, McLendon R, Rasheed BA, Yuan W, Kos I, Batinic-Haberle I, Jones S, Riggins GJ, Friedman H, Friedman A, Reardon D, Herndon J, Kinzler KW, Velculescu VE, Vogelstein B and Bigner DD (2009) IDH1 and IDH2 mutations in gliomas. N Engl J Med 360, 765–73. 19228619 10.1056/NEJMoa0808710 PMC2820383

[R92] Yuan Y, Shah N, Almohaisin MI, Saha S and Lu F (2021) Assessing fatty acid-induced lipotoxicity and its therapeutic potential in glioblastoma using stimulated Raman microscopy. Sci Rep 11, 7422. 33795756 10.1038/s41598-021-86789-9 PMC8016949

[R93] Yuen TI, Morokoff AP, Bjorksten A, D’Abaco G, Paradiso L, Finch S, Wong D, Reid CA, Powell KL, Drummond KJ, Rosenthal MA, Kaye AH and O’Brien TJ (2012) Glutamate is associated with a higher risk of seizures in patients with gliomas. Neurology 79, 883–9. 22843268 10.1212/WNL.0b013e318266fa89

[R94] Yuzawa S, Nishihara H, Yamaguchi S, Mohri H, Wang L, Kimura T, Tsuda M, Tanino M, Kobayashi H, Terasaka S, Houkin K, Sato N and Tanaka S (2016) Clinical impact of targeted amplicon sequencing for meningioma as a practical clinical-sequencing system. Modern Pathology 29, 708–716. 27102344 10.1038/modpathol.2016.81

[R95] Zhong Z, Wang Z, Wang Y, You G and Jiang T (2015) IDH1/2 mutation is associated with seizure as an initial symptom in low-grade glioma: A report of 311 Chinese adult glioma patients. Epilepsy Res 109, 100–5. 25524848 10.1016/j.eplepsyres.2014.09.012

[R96] Zhou L, Wang Z, Hu C, Zhang C, Kovatcheva-Datchary P, Yu D, Liu S, Ren F, Wang X, Li Y, Hou X, Piao H, Lu X, Zhang Y and Xu G (2019) Integrated Metabolomics and Lipidomics Analyses Reveal Metabolic Reprogramming in Human Glioma with IDH1 Mutation. J Proteome Res 18, 960–969. 30596429 10.1021/acs.jproteome.8b00663

